# Graphene Oxide Foam Supported Titanium(IV): Recoverable Heterogeneous Catalyst for Efficient, Selective Oxidation of Arylalkyl Sulfides to Sulfoxides Under Mild Conditions

**DOI:** 10.1038/s41598-017-07590-1

**Published:** 2017-08-03

**Authors:** Qinghe Wang, Wenxi Ma, Qiaolin Tong, Guijie Du, Jian Wang, Meng Zhang, Hailun Jiang, Huali Yang, Yongxiang Liu, Maosheng Cheng

**Affiliations:** 1 Key Laboratory of Structure-Based Drug Design and Discovery (Shenyang Pharmaceutical University), Ministry of Education, Shenyang, 110016 P. R. China; 2 Jinzhou Jiutai Pharmaceutical Co Ltd, Jinzhou, Liaoning China

## Abstract

An efficient and environmentally friendly method was designed for the oxidation of sulfides to sulfoxides with a recyclable, carbon-skeleton-based heterogeneous catalyst developed by titanium sulfate [Ti(SO_4_)_2_] mineralization on the surface of graphene oxide foam [Ti(SO_4_)_2_@GOF] by using 30 wt% H_2_O_2_ as oxidant. Several different substituted sulfides were examined to explore the scope of substrates of the selective oxidation. The excellent reusability and durability of Ti(SO_4_)_2_@GOF was demonstrated by recycling experiments and the catalyst was further applied in the preparation of pantoprazole sodium in a one-pot process.

## Introduction

The oxidation of sulfides to sulfoxides in a chemically selective manner is an important aspect of organic synthesis since sulfoxides are useful intermediates for the construction of various molecules^1–3^. The development of efficient and recyclable heterogeneous catalysts for the oxidation of sulfides to sulfoxides has been a research hotspot. Among various catalytic methods, the combination of various transition metals with organic skeletons to form recyclable catalyst system was widely studied^4^. The numerous ‘anchor sites’ on the surface of many different organic materials render the catalysts to be easily recovered, which helps to reduce environmental pollution^5^. The most frequently used supports include zeolite, resin and molecular sieves; however, vast resources consumption was required for these supports during the regenerative process. Graphene oxide (GO) and graphene oxide foam (GOF) are environmentally friendly and easily renewable materials which are attractive options for the organic skeletons or supports and widely used in heterogeneous catalysis^6^. The oxidation of sulfides to sulfoxides catalyzed by transition metals such as Mo, V, Fe, Zn, Cu and Co with different supports is a well-established method^7–18^. Besides, the ionic liquid-based polyoxometalate salt, biocatalysis and photocatalysis were also applied in this oxidation process^19–21^. Although many methods were used for the oxidation of arylalkyl sulfides, the water-soluble titanium has received less attention. Ti(SO_4_)_2_@GOF has been proved a promising catalyst for the selective oxidation of benzyl alcohol in our previous research^22^. Moreover, most studies for the oxidation of sulfides were focused on the organic titanium, instead of inorganic titanium^23, 24^. Therefore, we intend to develop a green process for the oxidation of arylalkyl sulfides with water-soluble Ti(SO_4_)_2_@GOF.

The detailed synthetic process and characterizations of Ti(SO_4_)_2_@GOF were reported in our previous study, which included scanning electron microscope, energy dispersive spectrometer, X-ray diffraction, solid-state NMR, thermal gravimetry analysis, infrared and BET analysis^22^. To examine the activity of the Ti(SO_4_)_2_@GOF for the selective oxidation of arylalkyl sulfides, we initially used methylphenyl sulfide as a model substrate. Furthermore, the recycling experiments proved that the Ti(SO_4_)_2_@GOF possessed excellent reusability and durability. Next, several different substituted sulfides were screened to explore the substrate scopes of this method. Finally, the industrial perspective of Ti(SO_4_)_2_@GOF was demonstrated by the preparation of pantoprazole sodium in a one-pot procedure.

## Results and Discussion

**Figure 1:**
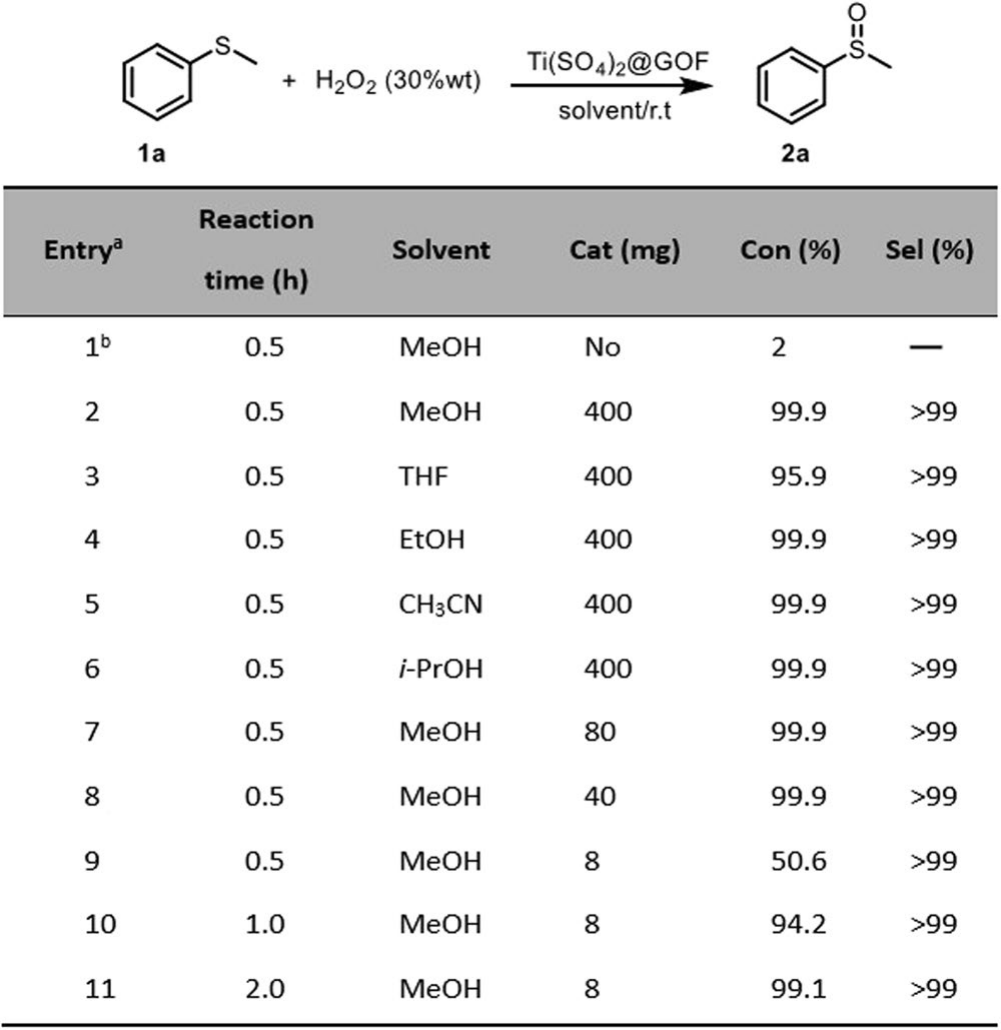
Oxidation of methylphenyl sulfide in different conditions. Reaction conditions: methylphenyl sulfide (800 mg, 6.45 mmol), solvent (10 mL) and 30 wt% H_2_O_2_ (0.74 mL, 6.5 mmol) was slowly dropped into reaction system. ^b^Were carried out with no Ti(SO_4_)_2_@GOF. The 2a was purified by chromatography on silica gel (isolated yield: 98%).

As shown in Fig. 1, preliminary screening experiments were performed under different reaction conditions with methylphenyl sulfide as the model substrate. The blank experiment showed that the yield of the sulfoxide was only 2% in the absence of catalyst (Fig. 1, entry 1). The reaction catalyzed by Ti(SO_4_)_2_@GOF was completed in a short time with high yield and selectivity (Fig. 1, entries 2–6). To further investigate the catalytic activity of the Ti(SO_4_)_2_@GOF catalyst, the oxidation of methylphenyl sulfide was performed in the presence of different amounts of the Ti(SO_4_)_2_@GOF. As illustrated in entries 7–11 in Fig. 1, the reaction was completed in 2 hours with 1% loading weight of Ti(SO_4_)_2_@GOF (Fig. 1, entry 11). The results demonstrate that the Ti(SO_4_)_2_@GOF has a high catalytic activity for the oxidation of methylphenyl sulfide. Besides, the selectivity towards sulfoxide is not related to the loading of the catalyst and reaction rates. The reusability and durability of the Ti(SO_4_)_2_@GOF catalyst were also evaluated in the oxidation of methylphenyl sulfide. The results showed that there are no significant changes in the conversion and selectivity after 10 times recycles (Figure S1). It is worth mentioning that the mild condition of our oxidation prevents Ti(SO_4_)_2_@GOF from decomposition under harsh conditions^22^.

**Figure 2:**
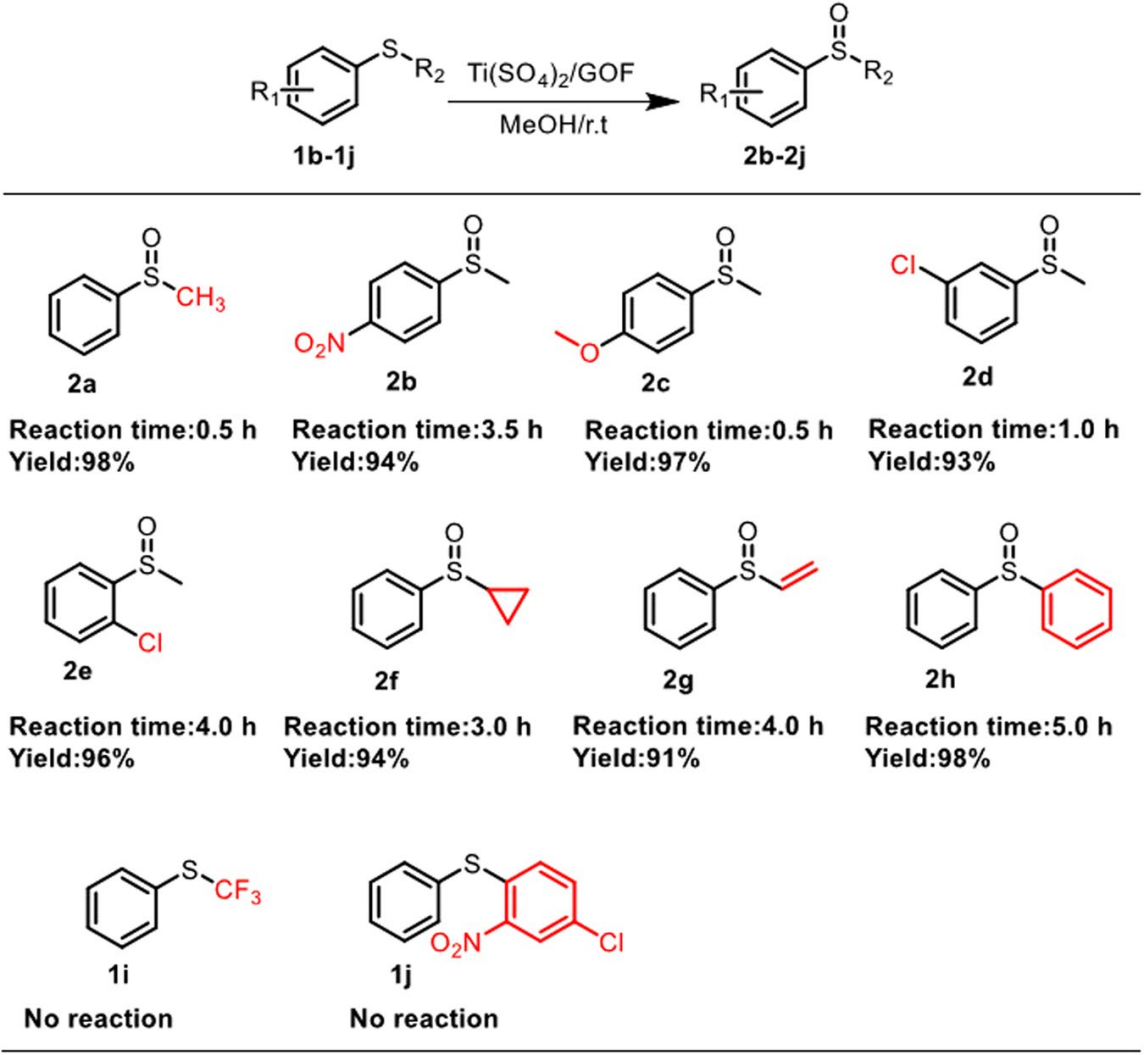
Oxidation of different substituted aryalkyl sulfides. Reaction conditions: arylalkyl sulfides (400 mg), MeOH (5 mL), Ti(SO_4_)_2_@GOF (20 mg), 1.01 eq. 30 wt% H_2_O_2_ was slowly dropped into reaction system.

Since Ti(SO_4_)_2_@GOF displayed a high catalytic activity for the selective oxidation of methylphenyl sulfide, it was deployed in the scope study under room temperature. As shown in Fig. 2, various substrates were successfully converted into their corresponding sulfoxides in high yields. Furthermore, the influences of electronic effects, steric hindrance and p-π conjunction of substitutes on reaction rates were studied according to the results of different substituted sulfides oxidation. The reaction rate of the substrate with electron-deficient phenyl group (**1b**) is much slower than that of the substrate with electron-rich phenyl group (**1c**). Subsequently, the **1d** and **1e** were screened to explore the effect of steric hindrance of the substitutes on benzene rings. The results demonstrated that the reaction rate of **1d** is significantly slower than that of **1e**. Furthermore, the reaction rates could also be affected by the alkyl moiety (**1f** and **1a**). On the other hand, the oxidation results of **1g** and **1h** indicated that the reaction rates would also be affected by the degree of p-π conjunction. It is important to notice that the oxidation of **1g** occurred at the sulfur atom, without affecting the C-C double bond. The reactions with chemically inert sulfides such as phenyltrifluoromethyl sulfide and 2-nitro-4-chlorodiphenyl sulfide proved unsuccessful (**1i** and **1j**).

**Figure 3:**
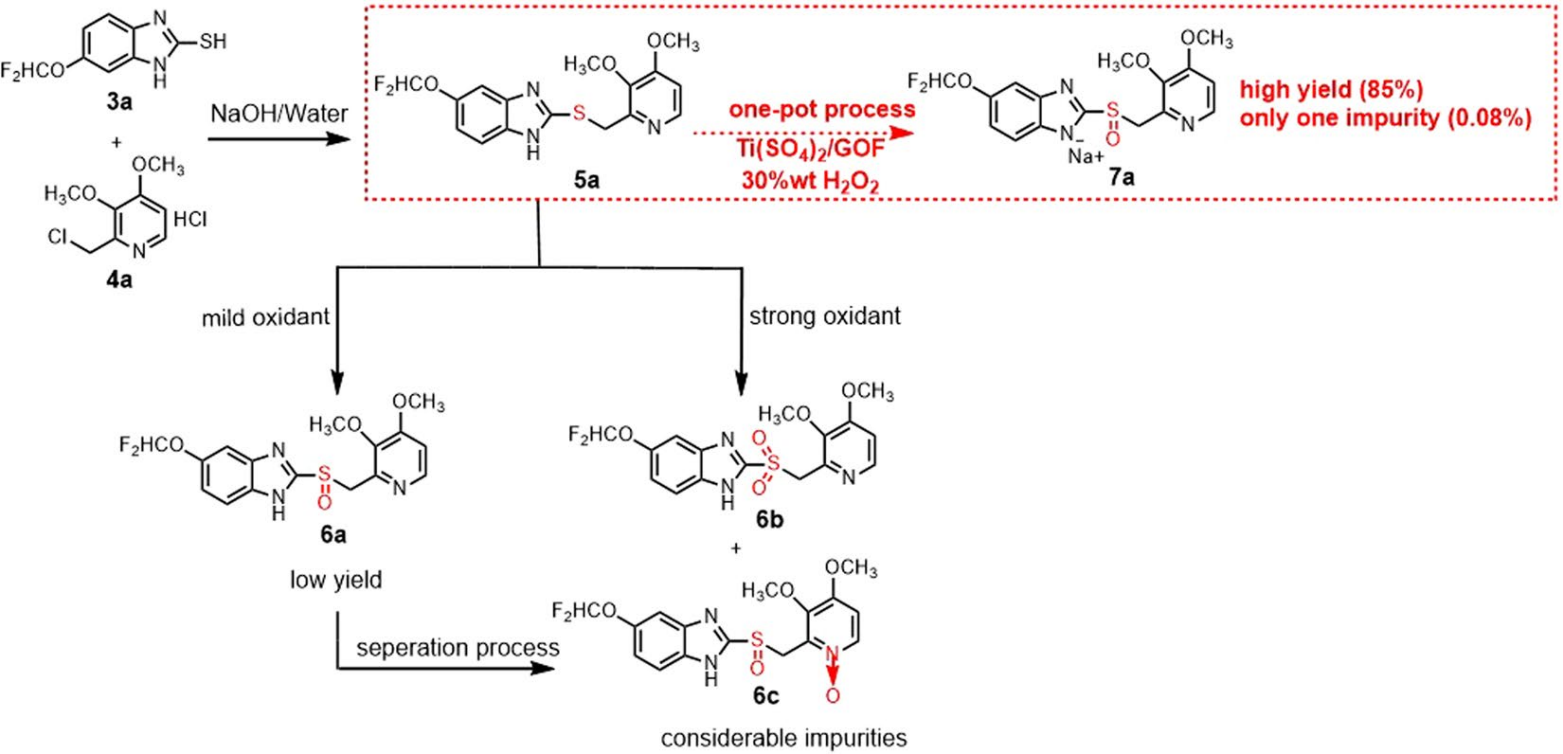
Process for the preparation of pantoprazole sodium.

The further application of Ti(SO_4_)_2_@GOF in the oxidation of arylalkyl sulfide was demonstrated by the preparation of the well-known proton pump inhibitor (PPI)-pantoprazole sodium, which is the third generation PPIs drug and is used to treat gastric acid-related diseases in clinic (Fig. 3)^25^. To test the reaction, the sulfide derivative **5a** was prepared by the condensation of thiol derivative **3a** and chloromethyl pyridine derivative **4a** in the presence of inorganic base. Traditionally, the oxidation of **5a** to **6a** was performed with peroxide such as *m*-chloroperoxy benzoic acid, hydrogen peroxide or *tert*-butyl hydroperoxide, etc., companied by the formation of considerable amounts of impurities, pantoprazole sulfone (**6b**) and pantoprazole *N*-oxide (**6c**)^26^. Both of them are very difficult to remove through simple workup process, especially **6b**, which is very similar to **6a** in physical and chemical properties. Alterative process for the oxidation of **5a** involves transition metals and hydrogen peroxide, in which the major drawback for this oxidation process is low conversion of **5a**. Moreover, it is necessary to isolate pantoprazole in the traditional oxidation process, which makes purification process very difficult owing to the instability of pantoprazole to various conditions (e.g., air, visible light, acid). In order to solve these problems in aforementioned oxidation process, the oxidation from **5a** to **6a** was performed by the Ti(SO_4_)_2_@GOF catalyst in a one-pot procedure, which avoided both the generation of impurities and the separation process. As a result, the one-pot synthesis of pantoprazole sodium was achieved with excellent results. The total yield was up to 85%, while the HPLC analysis demonstrates that there is only one impurity (**6b**) with 0.08% content. (HPLC analysis and other details were provided in Supporting Information).

## Methods

Ti(SO_4_)_2_@GOF was synthesized according to our previous reported procedures^22^. Other information including general information, general procedure for the oxidation of arylalkyl sulfide, recycling tests of Ti(SO_4_)_2_@GOF and one-pot process to synthesis pantoprazole sodium was provided in Supporting Information.

### Pantoprazole sodium and Arylalkyl sulfoxides^1^H and^13^C NMR analysis

White solid, purified by recrystallization in acetone (yield 85%).^1^H NMR (600 MHz, DMSO-*d*_6_) δ 8.23 (d, *J* = 5.63 Hz, 1H), 7.44 (d, *J* = 8.45 Hz, 1H), 7.24 (d, *J* = 1.97 Hz, 1H), 7.08 (d, *J* = 5.63 Hz, 1H), 7.05 (-CF_2_HO-, t, *J* = 75.75 Hz, 1H), 6.73 (dd, *J* = 8.45 Hz, *J* = 2.25 Hz, 1H), 4.72 (d, *J* = 12.67 Hz, 1H), 4.32 (d, *J* = 12.67 Hz, 1H), 3.90 (s, 3H), 3.78 (s, 3H); ^13^C NMR (150 MHz, DMSO-*d*_6_) δ 164.75 (s), 158.78 (s), 147.65 (s), 147.02 (s), 146.29 (s), 144.97 (s), 144.68 (s), 119.71 (s), 118.00 (s), 116.32 (s), 111.46 (s), 108.29 (s), 107.87 (s), 61.39 (s), 56.83 (s), 56.36 (s). HRMS: Tgt Mass 383.0751, found in 383.0753. Diff: −0.53 ppm.

### (Methylsulfinyl)benzene (2a)

White solid, purified by chromatography on silica gel (80% hexane in ethyl acetate). (isolated yield: 98%). ^1^H NMR (600 MHz, DMSO-*d*_6_) δ 7.68 (t, *J* = 7.2 Hz, 2H), 7.56 (dd, *J* = 6.6 Hz, 1.2 Hz, 2H), 7.54–7.52 (m, 1H), 2.73 (s, 3H); ^13^C NMR (150 MHz, DMSO-*d*_6_) δ 146.863 (s), 131.119 (s), 129.691 (s), 124.002 (s), 43.7040 (s). HRMS: Tgt Mass 140.02959, found in 140.02936, Diff: 1.59 ppm.

### 1-(Methylsulfinyl)-4-nitrobenzene (2b)

Yellow solid, purified by chromatography on silica gel (50% hexane in ethyl acetate) (isolated yield: 94.6%). ^1^H NMR (600 MHz, DMSO-*d*_6_) δ 8.40 (d, *J* = 8.73 Hz, 2H), 7.96 (d, *J* = 8.73 Hz, 2H), 2.84 (s, 3H); ^13^C NMR (150 MHz, DMSO-*d*_6_) δ 154.44 (s), 149.30 (s), 125.63 (s), 124.68 (s), 43.39 (s). HRMS: Tgt Mass 185.01466, found in 185.015, Diff: −1.84 ppm.

### 1-Methoxy-4-(methylsulfinyl)benzene (2c)

White solid, purified by chromatography on silica gel (50% hexane in ethyl acetate) (isolated yield: 97.8%). ^1^H NMR (600 MHz, DMSO-*d*_6_) δ 7.61 (d, *J* = 8.73 Hz, 2H), 7.12 (d, *J* = 8.80 Hz, 2H), 3.81 (s, 3H), 2.68 (s, 3H); ^13^C NMR (150 MHz, DMSO-*d*_6_) δ 161.69 (s), 137.73 (s), 125.97 (s), 115.19 (s), 55.95 (s), 43.79 (s). HRMS: Tgt Mass 170.04015, found in 170.03987, Diff: 1.63 ppm.

### 1-Chloro-2-(methylsulfinyl)benzene (2d)

Yellow oil, purified by chromatography on silica gel (50% hexane in ethyl acetate) (isolated yield: 96.7%). ^1^H NMR (600 MHz, DMSO-*d*_6_) δ 7.84 (d, *J* = 7.6 Hz, 1H), 7.64–7.67 (m, 1H), 7.58(dd, *J* = 4.8 Hz, 0.70 Hz, 2H), 2.79 (s, 3H); ^13^C NMR (150 MHz, DMSO-*d*_6_) δ 144.32 (s), 133.00 (s), 130.33 (s), 129.40 (s), 129.04 (s), 125.58 (s), 41.93 (s). HRMS: Tgt Mass 173.99061, found in 173.99054, Diff: 0.41 ppm.

### 1-Chloro-3-(methylsulfinyl)benzene (2e)

Yellow oil, purified by chromatography on silica gel (50% hexane in ethyl acetate) (isolated yield: 93.2%). ^1^H NMR (600 MHz, DMSO-*d*_6_) δ 7.74 (s, 1H), 7.63–7.66 (m, 1H), 7.60 (dd, *J* = 4.51 Hz, 0.99 Hz, 2H), 2.78 (s, 3H); ^13^C NMR (150 MHz, DMSO-*d*_6_) δ 149.39 (s), 134.49 (s), 131.59 (s), 131.01 (s), 123.79(s), 122.83 (s), 43.58 (s). HRMS: Tgt Mass 173.99061, found in 173.99098, Diff: −2.1 ppm.

### (Cyclopropylsulfinyl)benzene (2f)

Colorless oil, purified by chromatography on silica gel (60% hexane in ethyl acetate) (isolated yield: 94.1%). ^1^H NMR (600 MHz, DMSO-*d*_6_) δ 7.68 (d, *J* = 7.18 Hz, 2H), 7.53–7.58 (m, 3H), 2.42–2.46 (m, 1H), 0.96–1.01 (m, 1H), 0.89–0.93 (m, 2H), 0.79–0.83 (m, 1H); ^13^C NMR (150 MHz, DMSO-*d*_6_) δ 145.73 (s), 131.23 (s), 129.63 (s), 124.34 (s), 33.32 (s), 3.27 (s), 2.13 (s). HRMS: Tgt Mass 166.04524, found in 166.04535, Diff: −0.71 ppm.

### (Vinylsulfinyl)benzene (2g)

Yellow oil, purified by chromatography on silica gel (70% hexane in ethyl acetate) (isolated yield: 91.2%). ^1^H NMR (600 MHz, DMSO-*d*_6_) δ 7.63 (dd, *J* = 8.73 Hz, *J* = 1.55 Hz, 2H), 7.53–7.59 (m, 3H), 6.98 (q, *J* = 16.33 Hz, *J* = 9.57 Hz, 1H), 6.04 (d, *J* = 16.33 Hz, 1H), 5.93 (d, *J* = 9.57 Hz, 1H); ^13^C NMR (150 MHz, DMSO-*d*_6_) δ 144.15 (s), 144.07 (s), 131.47 (s), 129.95 (s), 124.72 (s), 120.19 (s). HRMS: Tgt Mass 152.02959, found in 152.02973, Diff: −0.96 ppm.

### Sulfinyldibenzene (2h)

White solid, purified by chromatography on silica gel (60% hexane in ethyl acetate) (isolated yield: 98.7%). ^1^H NMR (600 MHz, DMSO-*d*_6_) δ 7.71 (d, *J* = 7.32 Hz, 4H), 7.47–7.53 (m, 6H); ^13^C NMR (150 MHz, DMSO-*d*_6_) δ 146.40 (s), 131.54 (s), 129.95 (s), 124.59 (s). HRMS: Tgt Mass 202.04524, found in 202.0448, Diff: 2.16 ppm.

## Conclusions

In summary, Ti(SO_4_)_2_@GOF showed a high catalytic activity and chemical selectivity for the oxidation of arylalkyl sulfides to sulfoxides using 30%wt H_2_O_2_ as oxidant. The recycling test of Ti(SO_4_)_2_@GOF proved that it possessed excellent reusability and could be recycled facilely. Moreover, the Ti(SO_4_)_2_@GOF works successfully for the oxidation of a broad range of substrates with excellent isolated yields. The catalyst was also applied in the one-pot preparation of pantoprazole sodium with an excellent yield.

## Electronic supplementary material

Supplementary Information
