# Supplementary material for: Graphene Oxide Foam Supported Titanium(IV): Recoverable Heterogeneous Catalyst for Efficient, Selective Oxidation of Arylalkyl Sulfides to Sulfoxides Under Mild Conditions
Source: Sci Rep. 2017 Aug 3;7:7209. doi: 10.1038/s41598-017-07590-1 (PMC5543132; doi:10.1038/s41598-017-07590-1)
Supplement: Supplementary file 1 — Supplementary Information [file 41598_2017_7590_MOESM1_ESM.doc]

**Supporting Information**

Graphene Oxide Foam Supported Titanium(IV): Recoverable Heterogeneous Catalyst for Efficient, Selective Oxidation of Arylalkyl Sulfides to Sulfoxides Under Mild Conditions

Qinghe Wang^a,1^, Wenxi Ma^a,1^, Qiaolin Tong^a^, Guijie Du^b^, Jian Wang^a^, Meng Zhang^a^, Hailun Jiang^a^, Huali Yang^a^, Yongxiang Lui*^a^ & Maosheng Cheng*^a^

*^a^ Key Laboratory of Structure-Based Drug Design and Discovery (Shenyang Pharmaceutical University), Ministry of Education, Shenyang 110016, P. R. China*

*^b^ Jinzhou Jiutai Pharmaceutical Co Ltd, Jinzhou, Liaoning, China.*

**Table of content**

1. **General Information…………………………………………....…S2**
2. **General procedure for oxidation of arylalkyl sulfides…………..S2**
3. **Recycling tests of Ti(SO_4_)_2_@GOF...................................................S2**
4. **One-pot process to synthesis pantoprazole sodium……………...S3**
5. **HPLC analysis for pantoprazole sodium…………………………S4**
6. **^1^H and ^13^C spectra of arylalkyl sulfoxides………………………..S5**
7. **General Information**

All reactions were carried out under different conditions in round-bottom flask. All solvents and reactants were directly bought from commercial sources without further purification. For [column chromatograph](http://www.baidu.com/link?url=oc_qtF2uog9iiZi4UM4r2RkDvgoIg-LZ_eo4UUMSb6LVOujwq5gy-pxQJwt-5fKjHvjZeOrVs6JRDh_i3ER_u0Hfcoo2GxWPQ2ImnrpvPeZAsNYT3-LgG0JbjDJ3tkj2), silica gel GF254 used. ^1^H-NMR- and ^13^C-NMR-spectra were recorded on Bruker Avance 600 (^1^H: 600 MHz; ^13^C: 150 MHz).For ^1^H-NMR and ^13^C-NMR, CD_3_SOCD_3_ used as solvents and tetramethylsilane (TMS) as internal standard. Chemical shits are given in parts per million (ppm). The abbreviations s, d, t, q, and m stand for singlet, doublet, triplet, quartet, and multiplet. High resolution mass spectra (HRMS) were obtained on Agilent Technologies 6530 Accurate-Mass Q-TOF LC/MS (ESI with AJS). The selectivity and conversion in oxidation of methylphenyl sulfide was determined on HPLC with areas of peak normalization method (pump: waters 1525, detector: waters 2489, chromatographic column: WondaSil C-18, monitoring wavelength used 254nm).

**2. General procedure for oxidation of arylalkyl sulfides**

The reactions were carried out with sulfide 400 mg, 5 mL solvent and 20 mg Ti(SO_4_)_2_@GOF added into round-bottom flask followed by stirred 5 minutes to ensure Ti(SO_4_)_2_@GOF well-dispersed in solvent. Next, slowly dropped 30wt% H_2_O_2_ (1.01 eq) at room temperature. Monitored reaction process by HPLC (Mobile phase: acetonitrile/water=3/7). After complete the reaction, the Ti(SO_4_)_2_@GOF was removed by filtration. The corresponding sulfoxide was isolated by [column chromatograph](http://www.baidu.com/link?url=oc_qtF2uog9iiZi4UM4r2RkDvgoIg-LZ_eo4UUMSb6LVOujwq5gy-pxQJwt-5fKjHvjZeOrVs6JRDh_i3ER_u0Hfcoo2GxWPQ2ImnrpvPeZAsNYT3-LgG0JbjDJ3tkj2).

**3. Recycling tests of Ti(SO_4_)_2_@GOF**

The oxidation was carried out under identical reaction conditions as described in the general procedure for the oxidation of sulfides. After the completion of each run, the Ti(SO_4_)_2_@GOF was removed by filtration and washed by CH_2_Cl_2_ (3×20 mL), dried and reused in the following run.

**Figure S1.** Recycling tests of Ti(SO_4_)_2_@GOF for oxidation of methylphenyl sulfide*^a^*


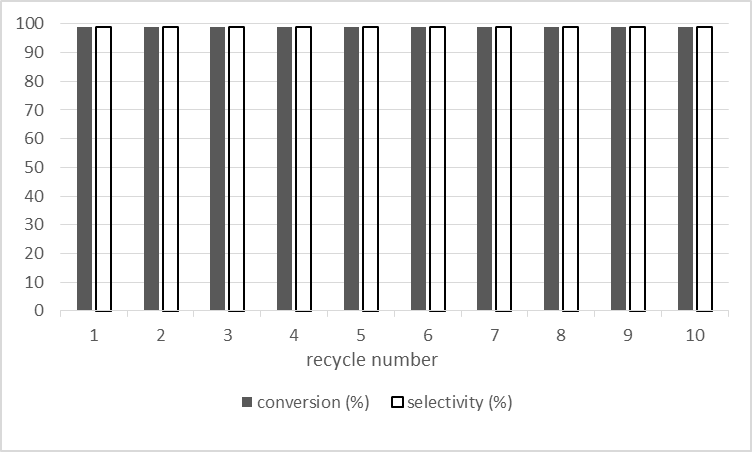


*^a^*reactions condition: methylphenyl sulfide (400 mg, 3.2 mmol), Ti(SO_4_)_2_@GOF (40 mg), MeOH (5 mL), 30wt% H_2_O_2_ (0.37 mL, 3.25 mmol).

**4. One-pot process to synthesis pantoprazole sodium**

Pantoprazole sulfide (**5a**) 6.11 g and 0.24 g Ti(SO_4_)_2_@GOF dissolved into 60 mL MeOH stirred 10 minutes under 0^o^С. Next, the 180 mg 30wt% H_2_O_2_ was slowly dropped into reaction system followed by rise temperature to 25^o^С. Monitored reaction process by TLC. After completion of the oxidation, the MeOH was removed by reduced pressure distillation, then added 30 mL acetone into bottom. Next, the Ti(SO_4_)_2_@GOF was removed by filtration and washed 3 times by acetone. Then combined the filtrate and the NaOH solution (1 g/mL) was dropped into reaction system under 35^o^C then cool to afford white solid as pantoprazole sodium. Filtration to obtain pantoprazole sodium (**7a**)，total yield 85%. It should be pointed out that the pantoprazole (**6a**) was not be isolated in the whole process. Impurities determined by HPLC (WondaSil C-18). Mobile phase A: (1.74 g/L solution of dipotassium hydrogen phosphate adjusted to pH 7.00 ± 0.05 with a 330 g/L solution of phosphoric acid). Mobile phase B: acetonitrile for chromatography. A:B=67:33, UV=289 nm, Flow rate=1.0 mL/min, rt=15.18 min.

**5. HPLC analysis for pantoprazole sodium**


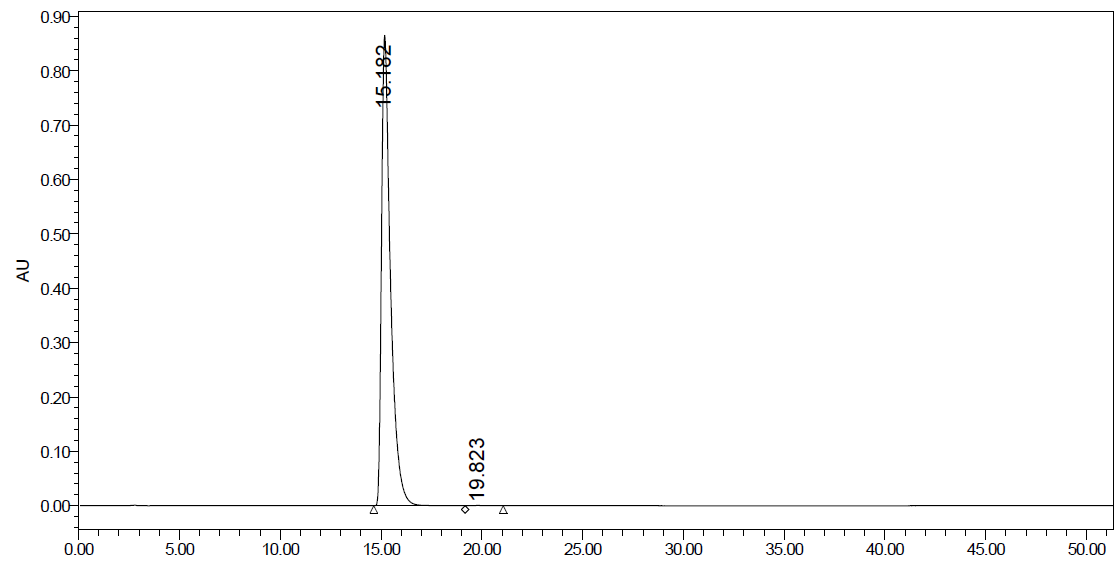


| RT (time) | Peak Area | Area% |
| --- | --- | --- |
| 15.18 min | 26855627 | 99.92 |
| 19.82 min | 20768 | 0.08 |

**6. ^1^H and ^13^C spectra of arylalkyl sulfoxides**

**
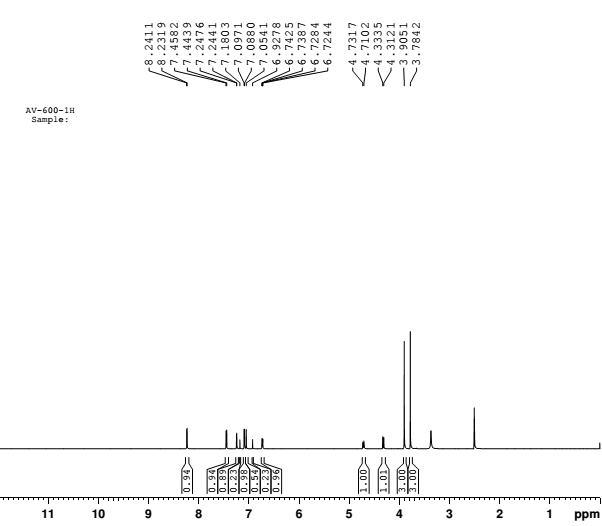
**

^1^H NMR Spectrum of Pantoprazole sodium


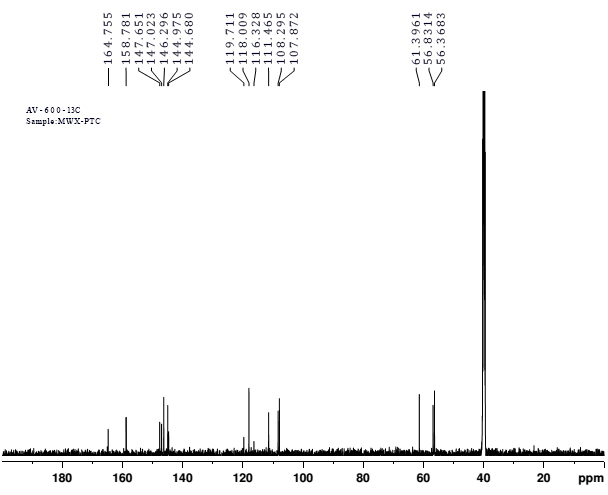


^13^C NMR Spectrum of Pantoprazole sodium

**
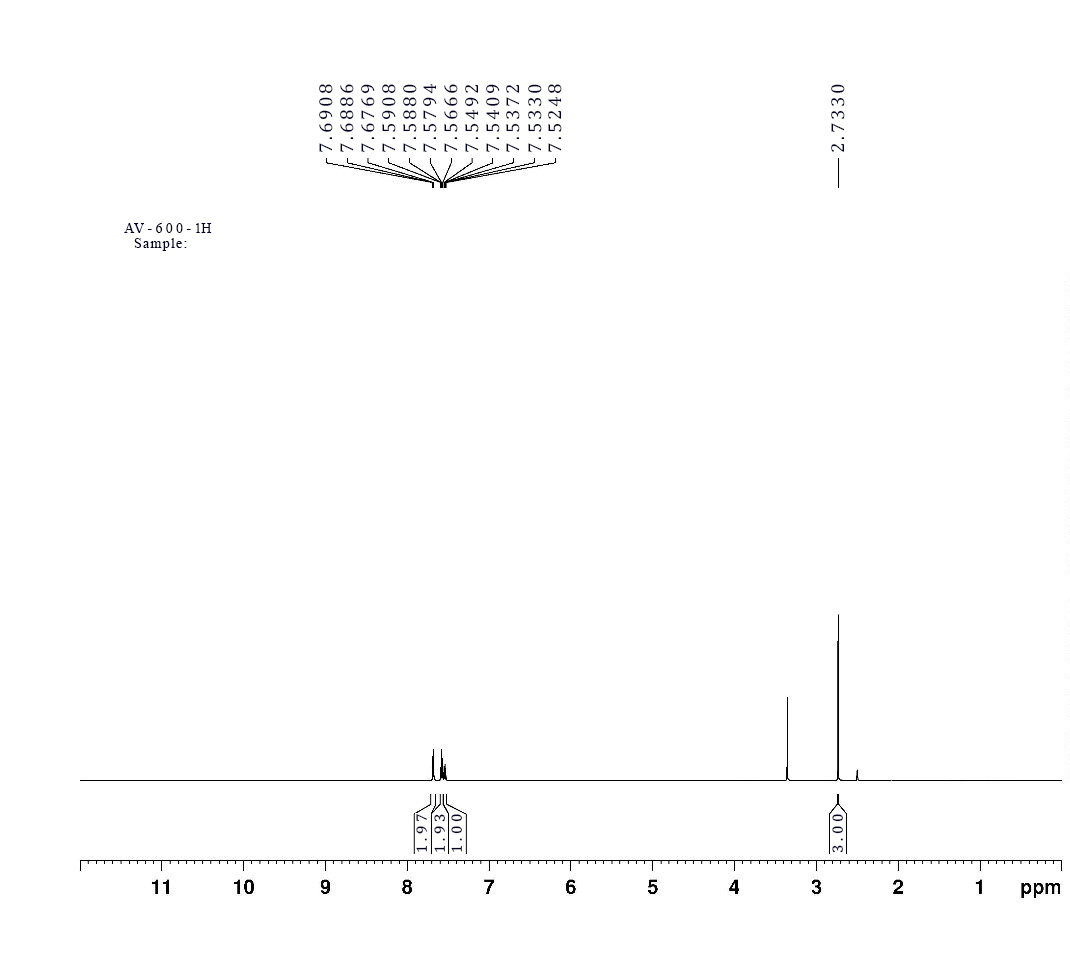
**

^1^H NMR Spectrum of (methylsulfinyl)benzene

**
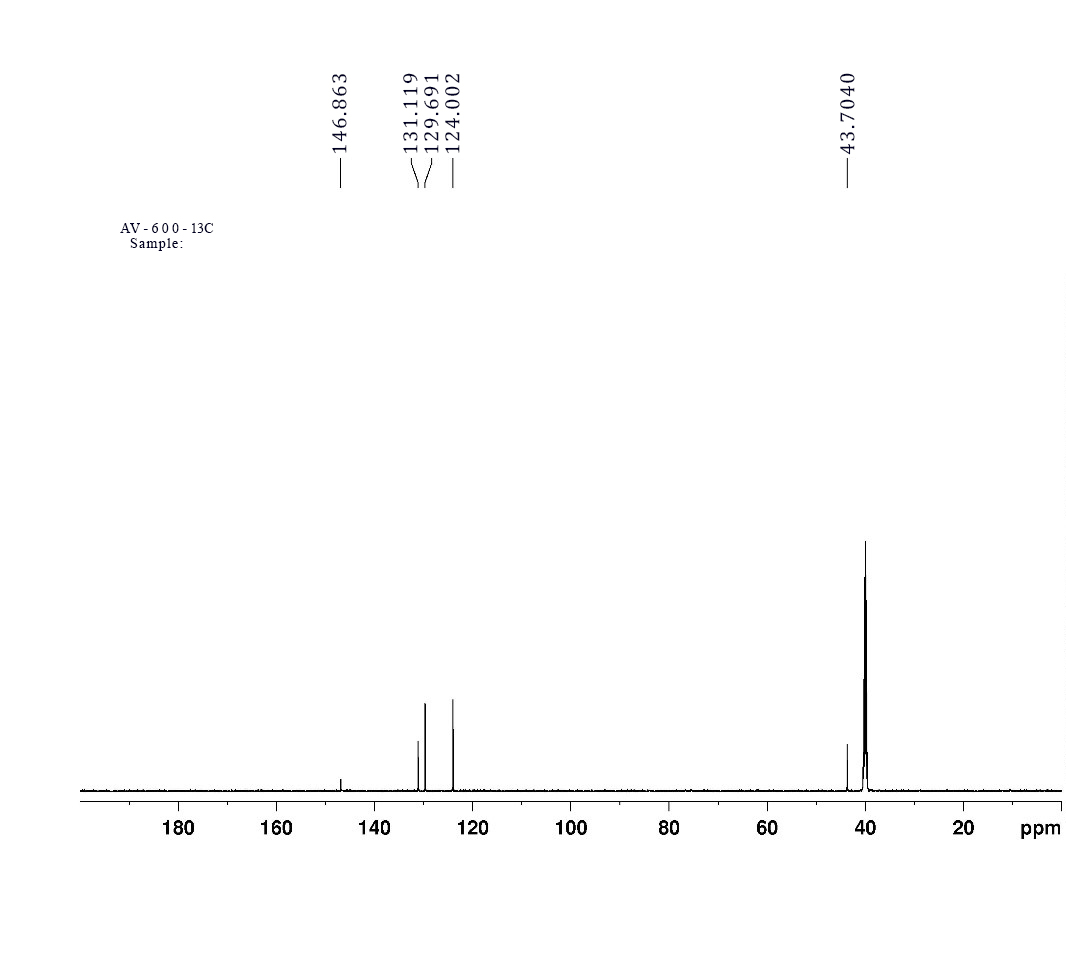
**

^13^C NMR Spectrum of (methylsulfinyl)benzene

**
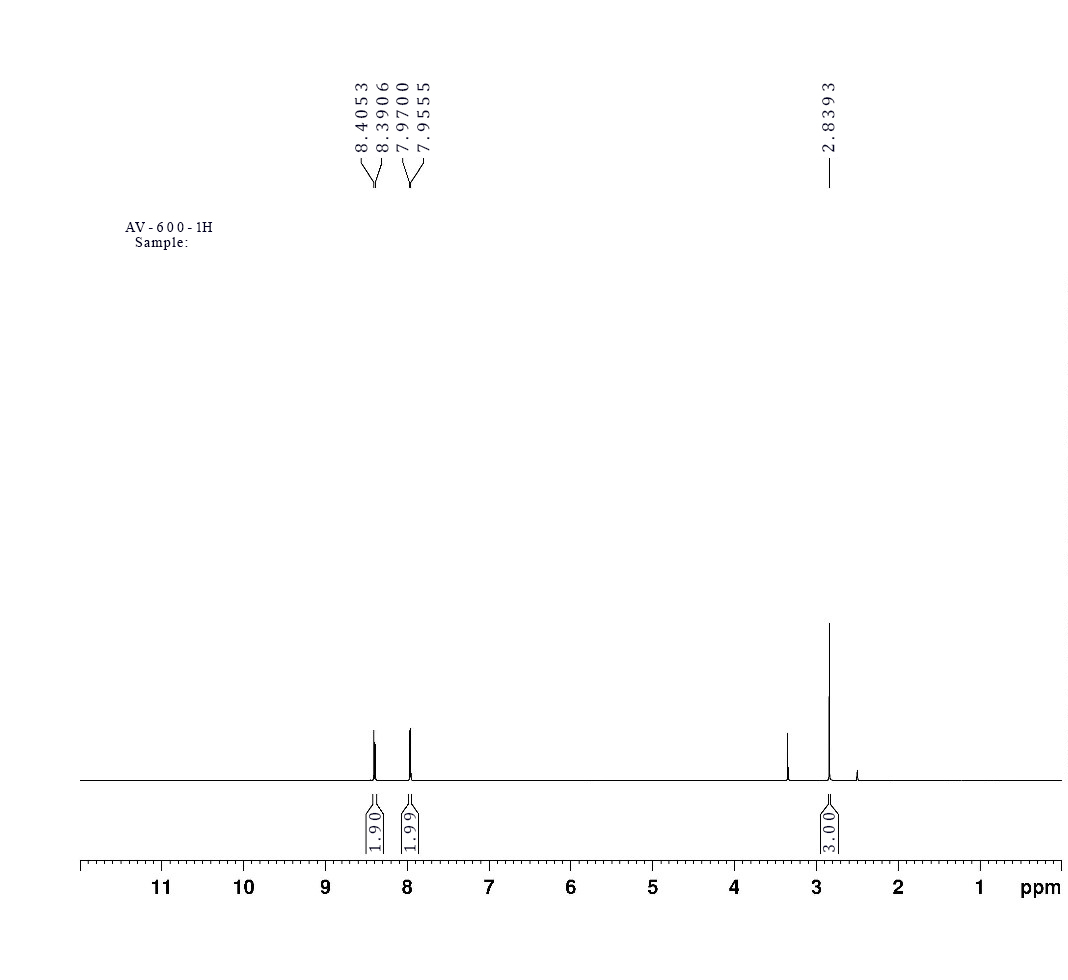
**

^1^H NMR Spectrum of 1-(methylsulfinyl)-4-nitrobenzene

**
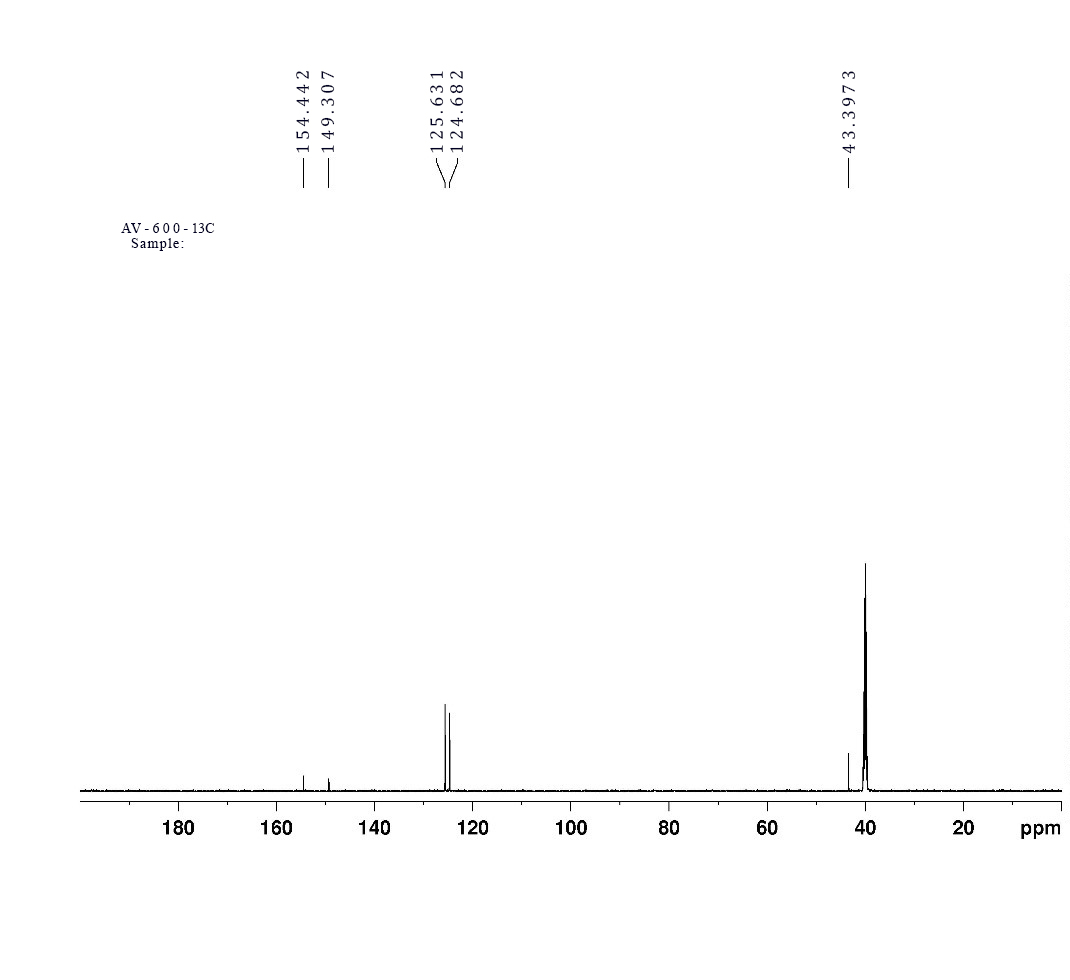
**

^13^C NMR Spectrum of 1-(methylsulfinyl)-4-nitrobenzene


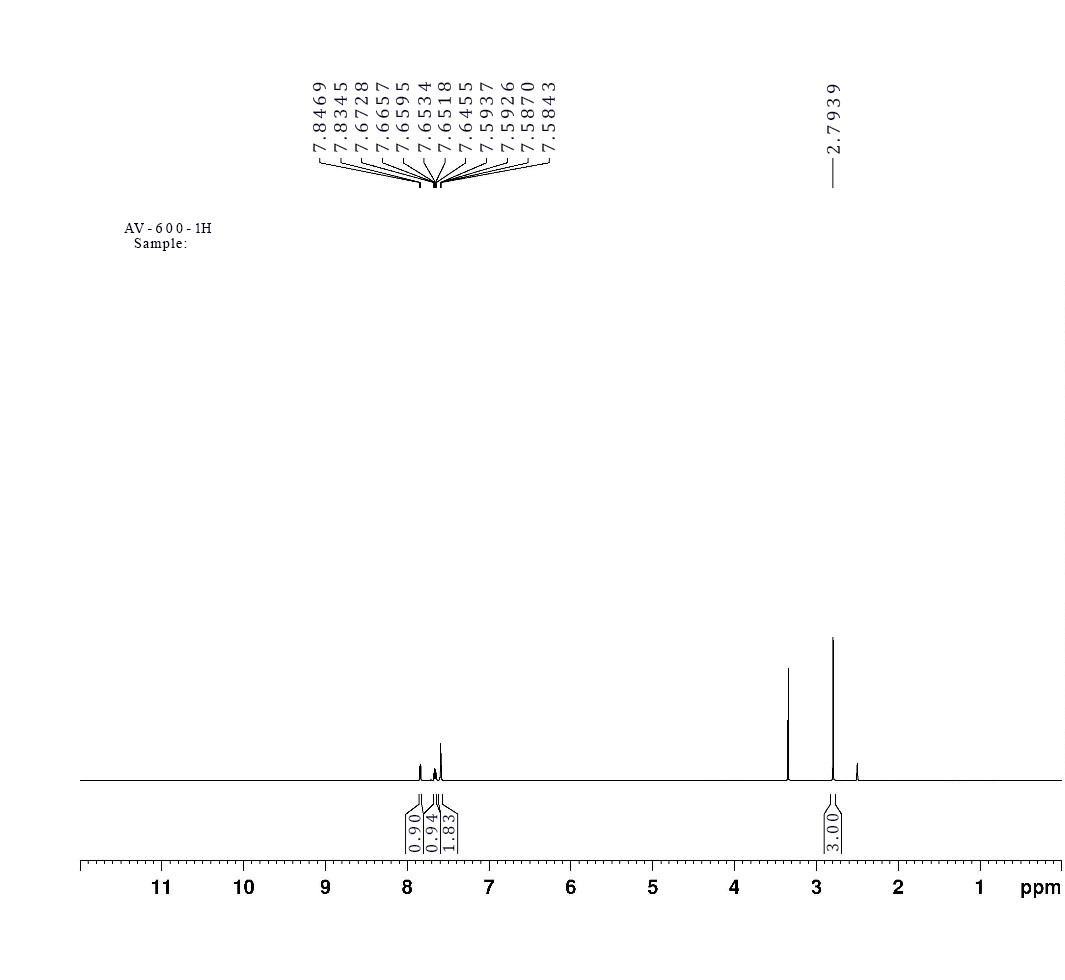


^1^H NMR Spectrum of 1-chloro-2-(methylsulfinyl)benzene


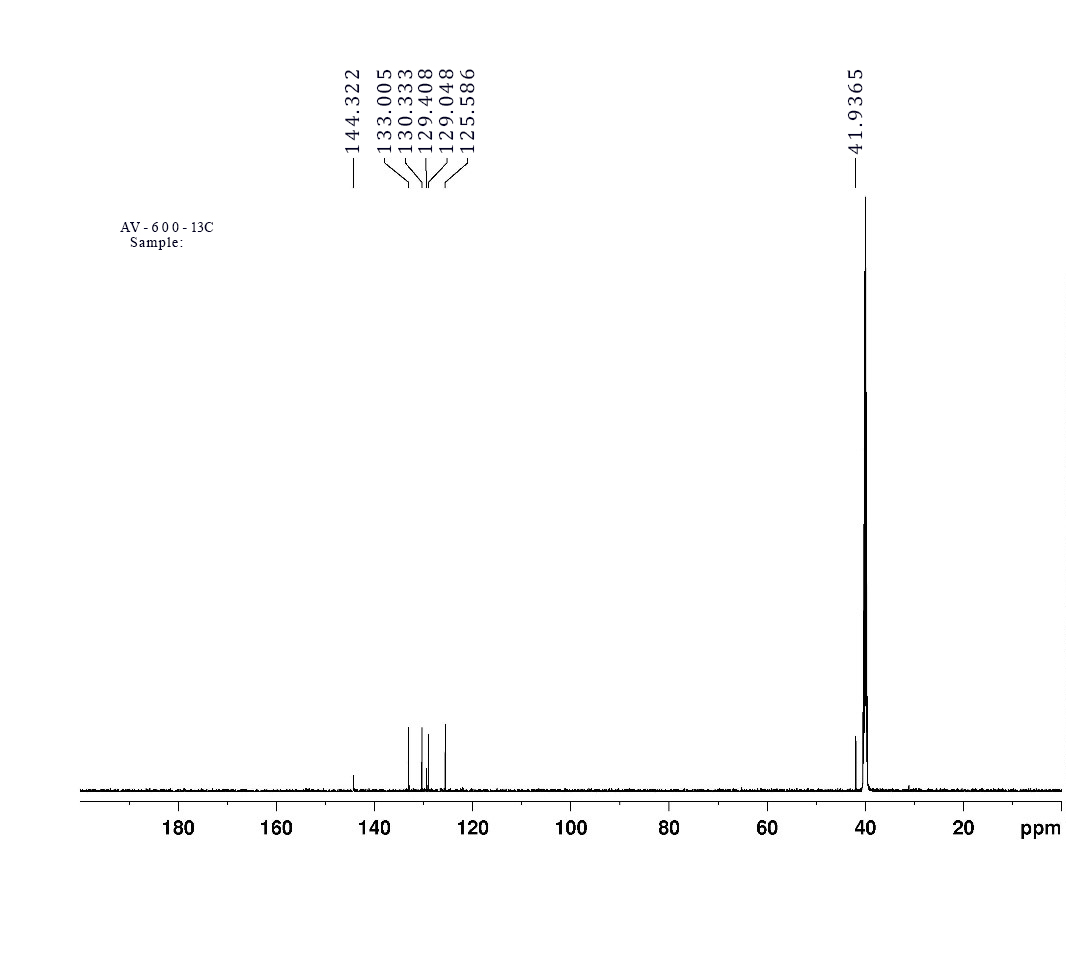


^13^C NMR Spectrum of 1-chloro-2-(methylsulfinyl)benzene


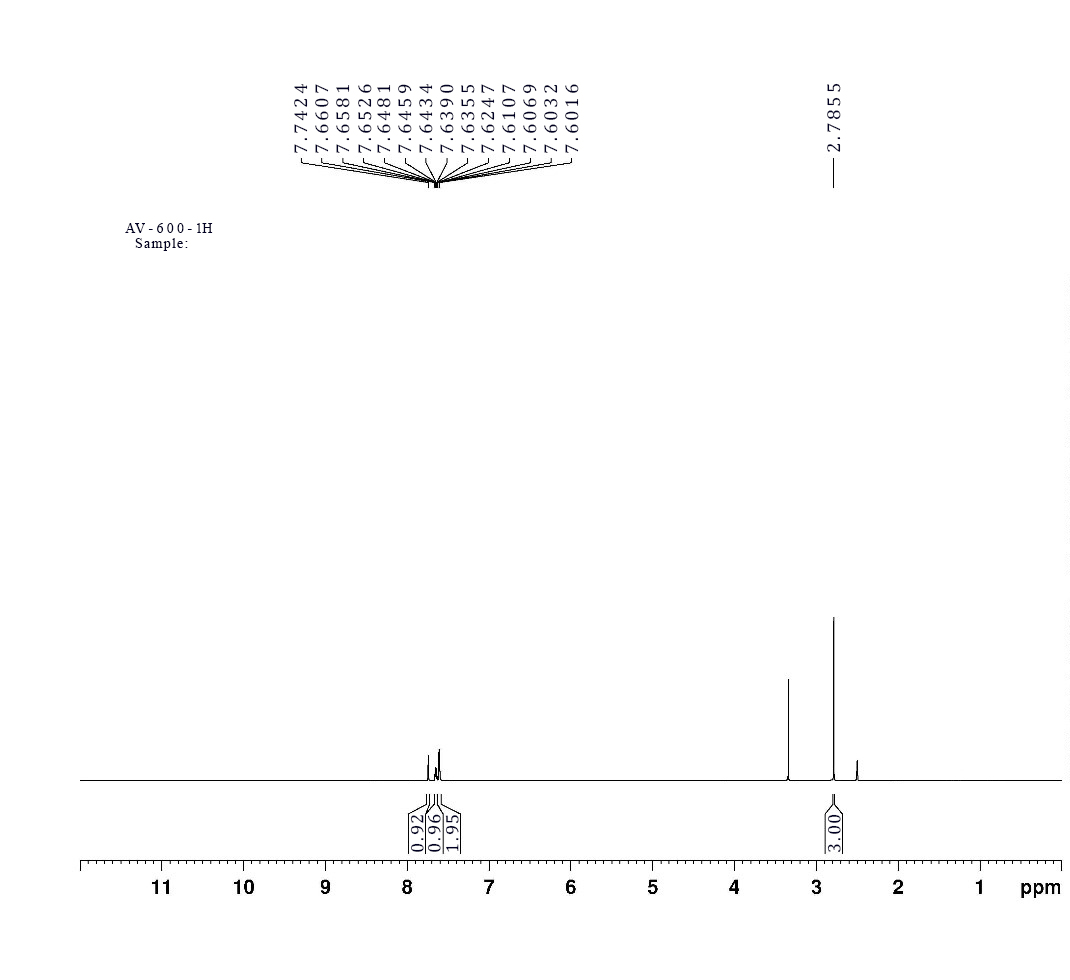


^1^H NMR Spectrum of 1-chloro-3-(methylsulfinyl)benzene


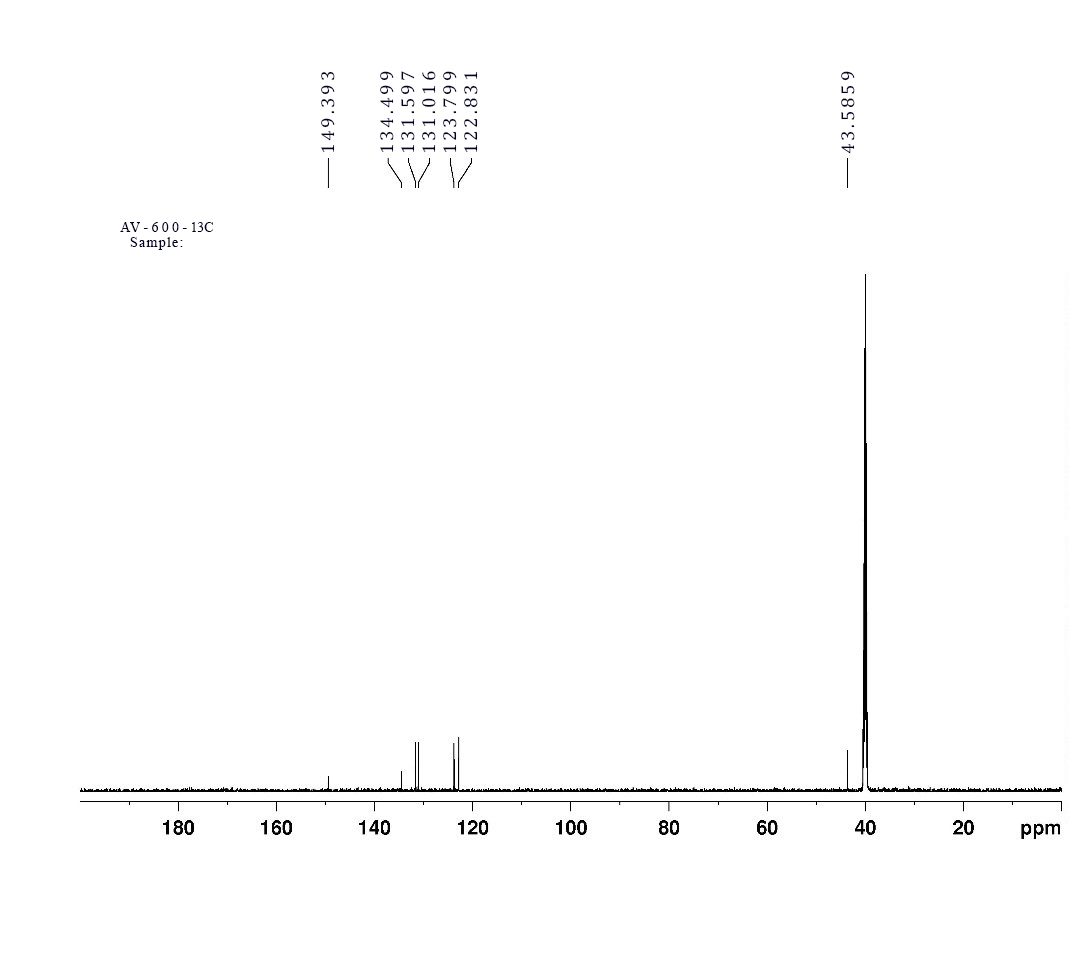


^13^C NMR Spectrum of 1-chloro-3-(methylsulfinyl)benzene


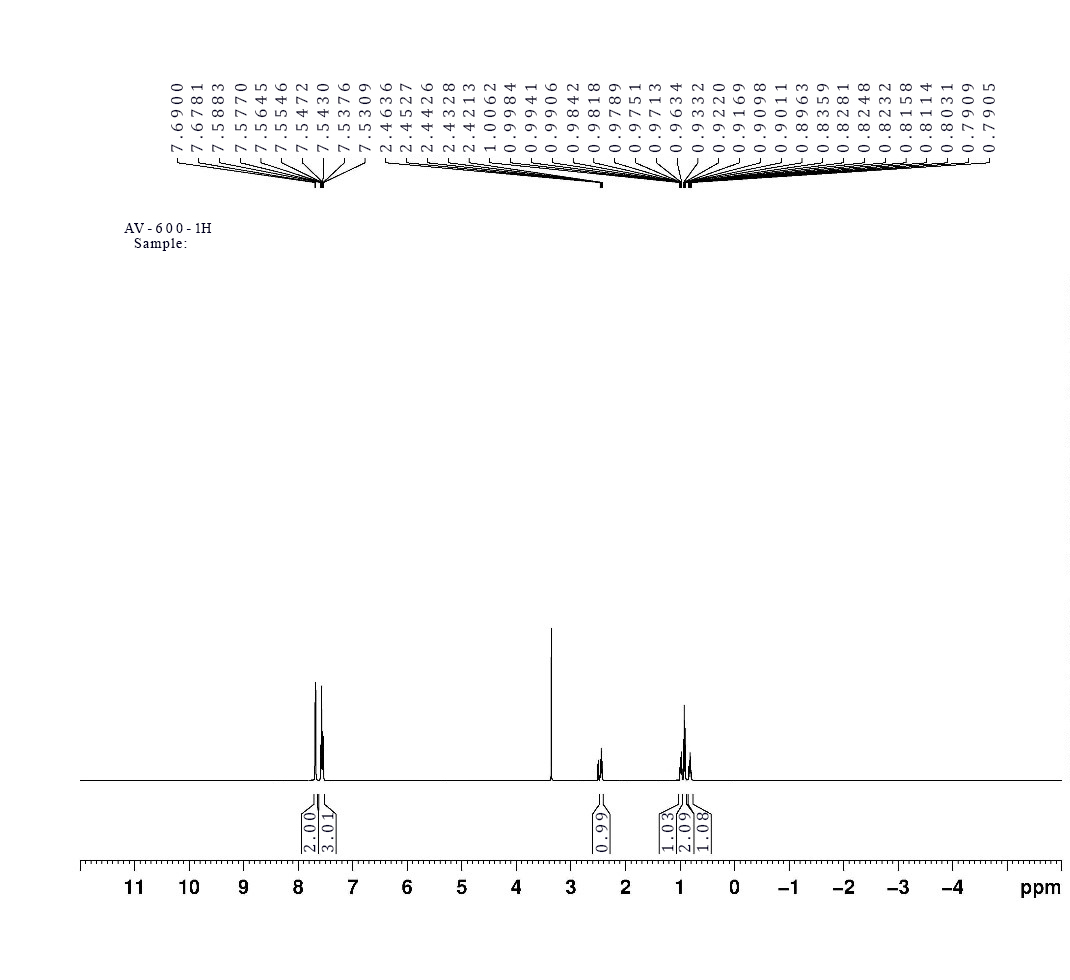


^1^H NMR Spectrum of (cyclopropylsulfinyl)benzene


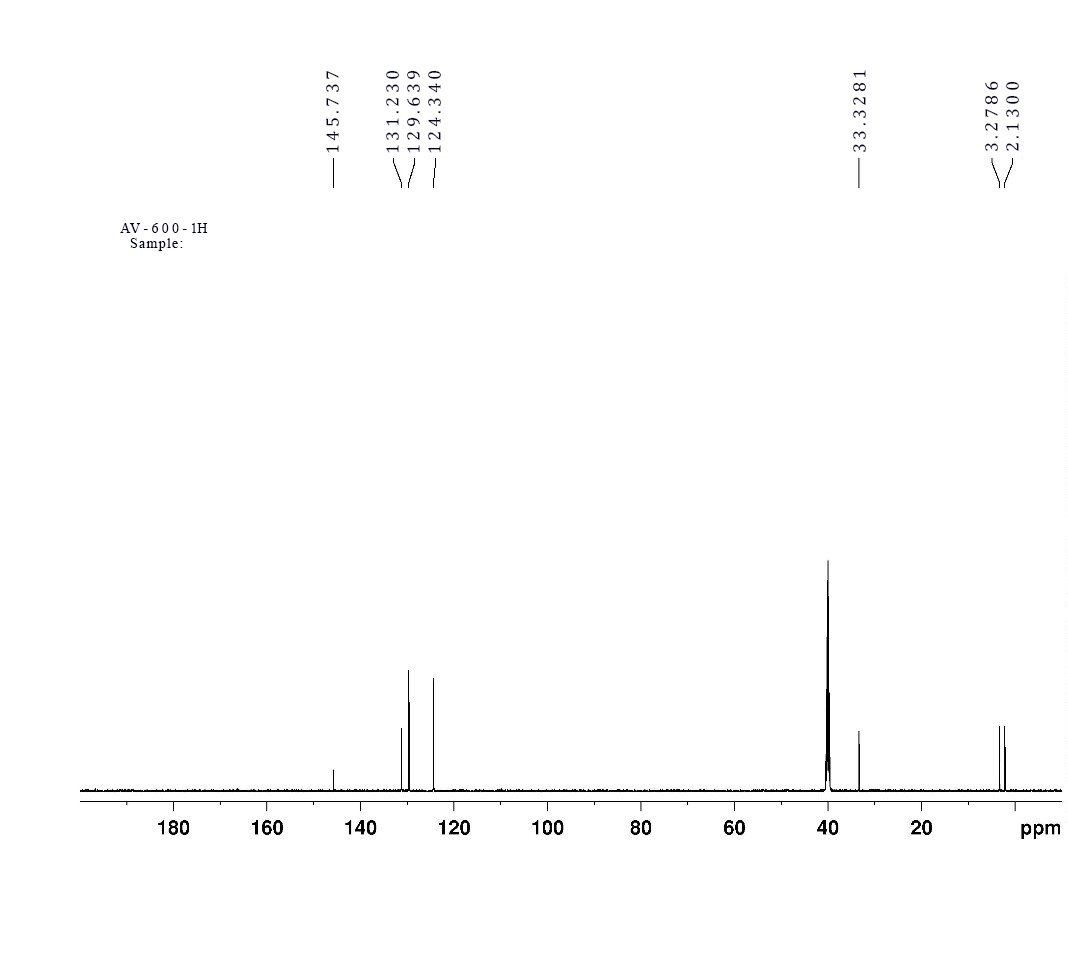


^13^C NMR Spectrum of (cyclopropylsulfinyl)benzene


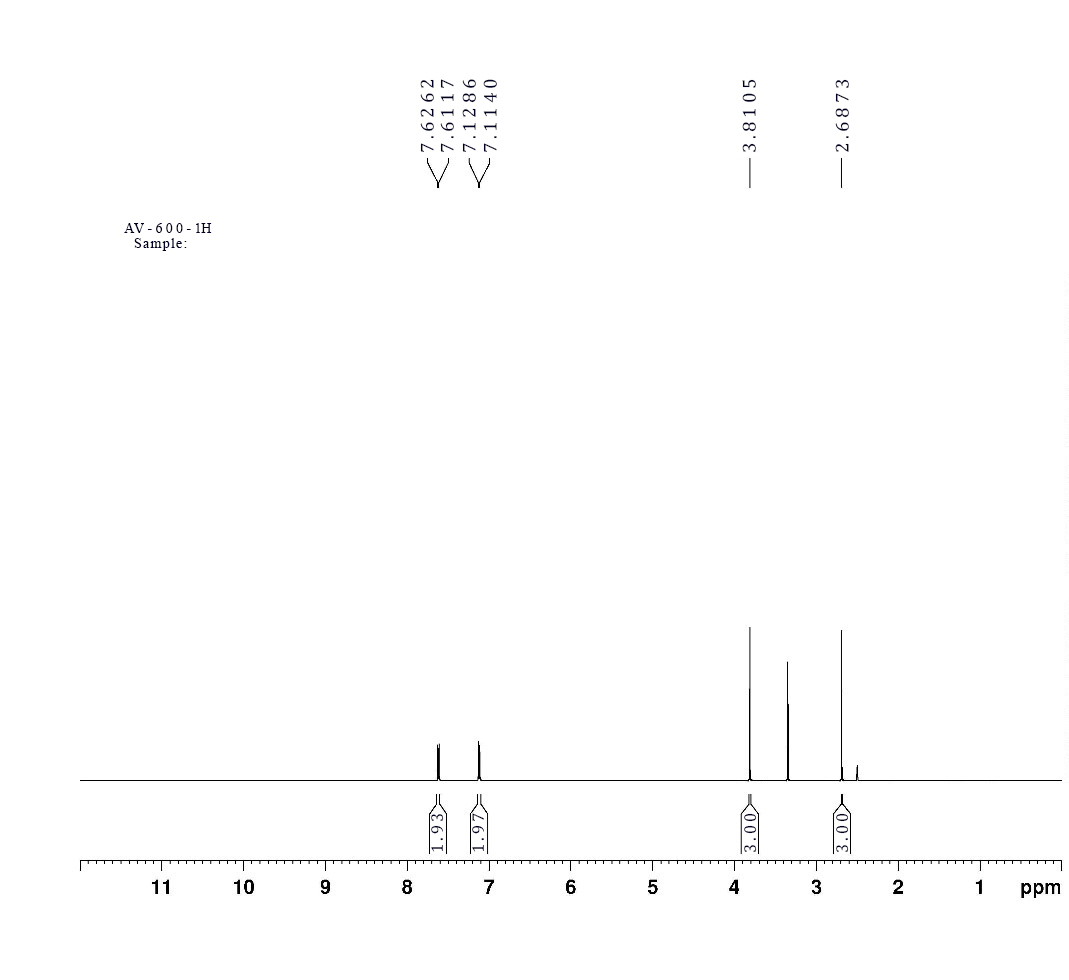


^1^H NMR Spectrum of 1-methoxy-4-(methylsulfinyl)benzene


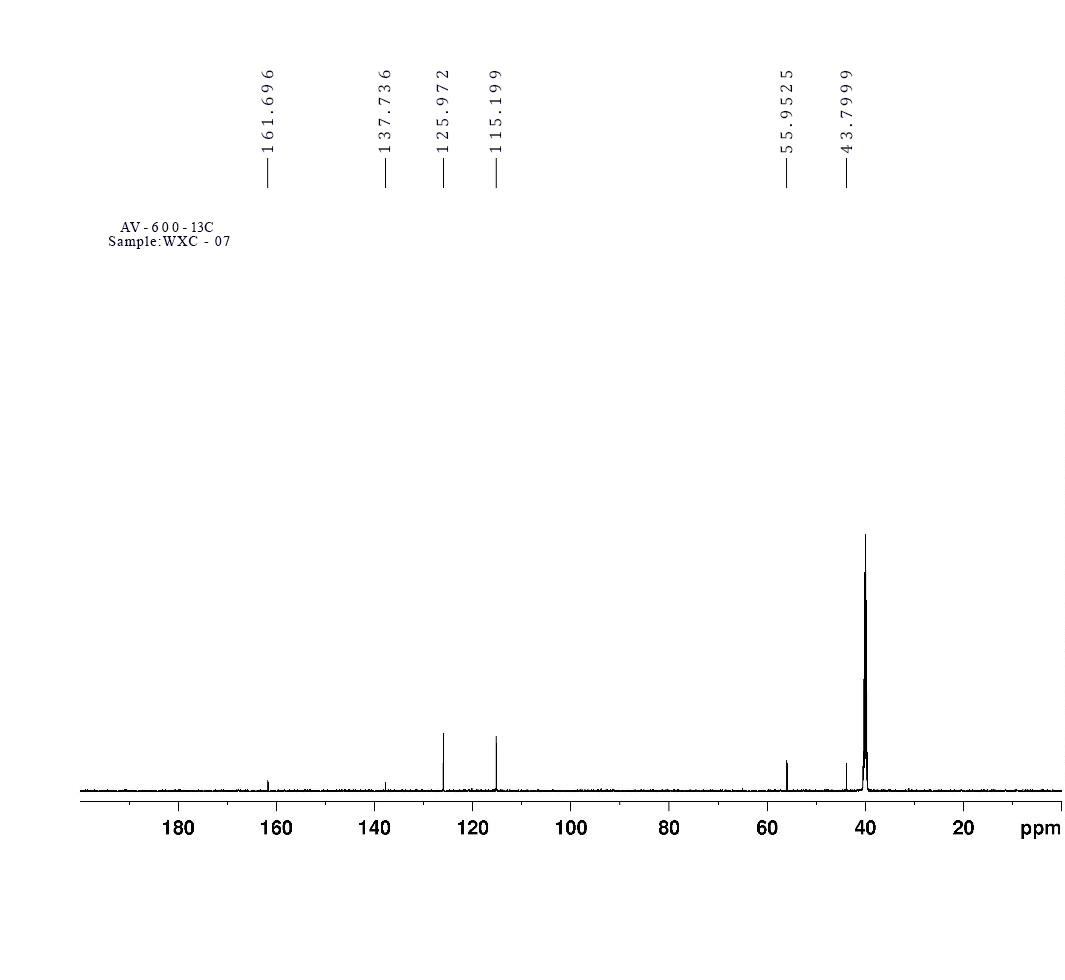


^13^C NMR Spectrum of 1-methoxy-4-(methylsulfinyl)benzene


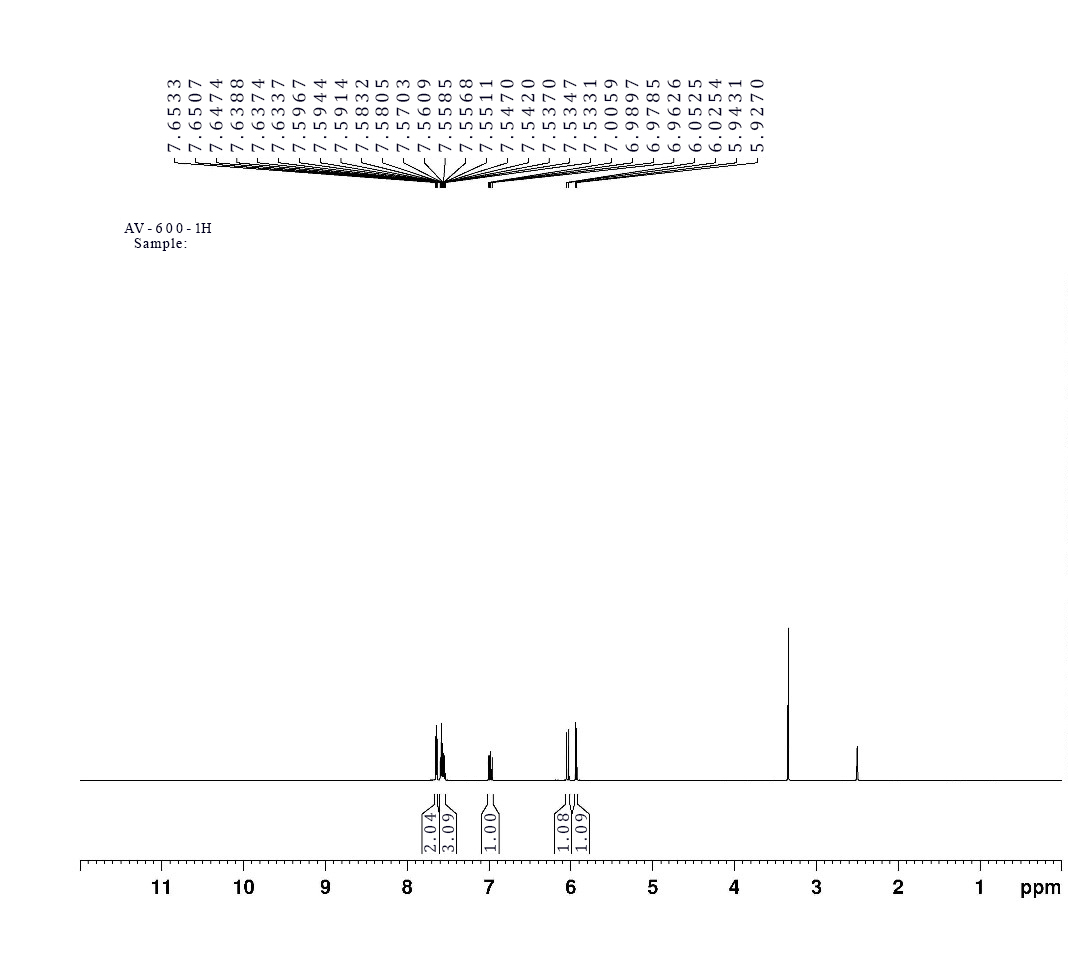


^1^H NMR Spectrum of (vinylsulfinyl)benzene


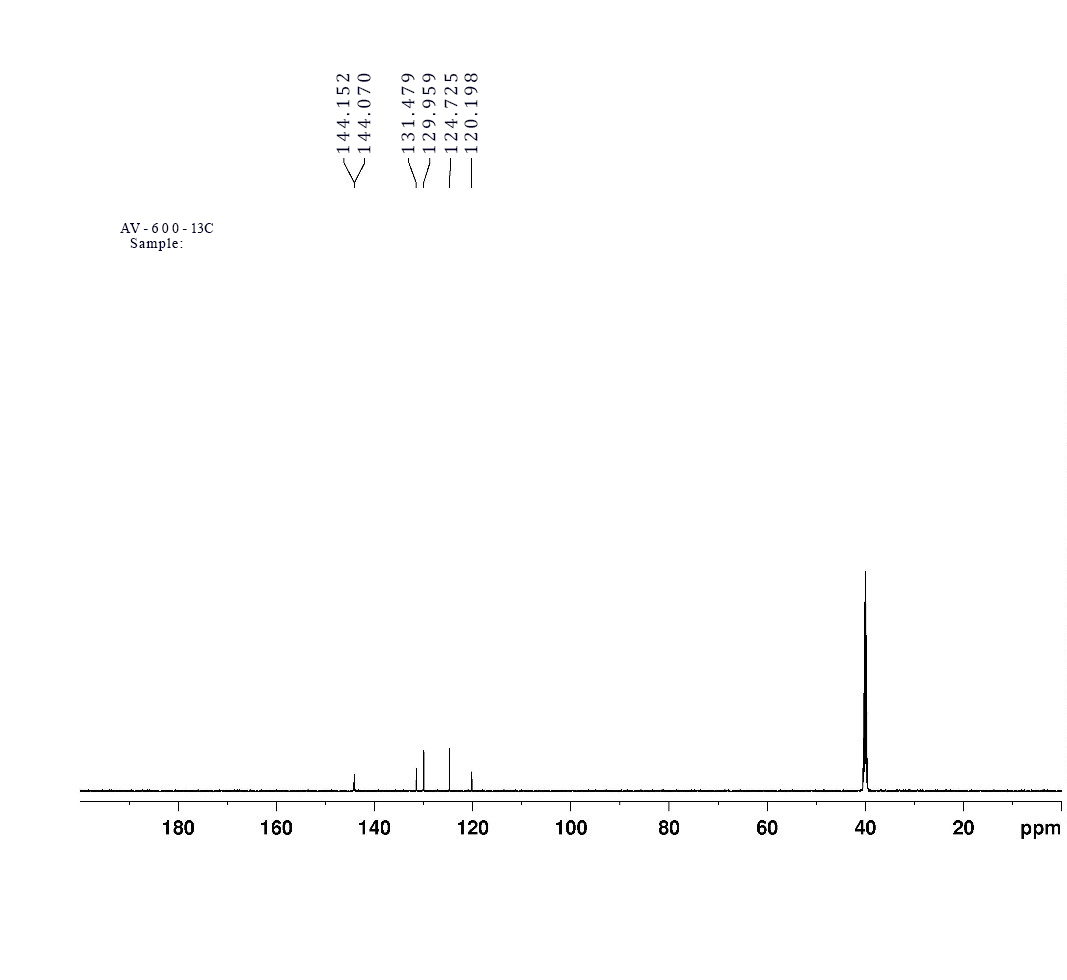


^13^C NMR Spectrum of (vinylsulfinyl)benzene


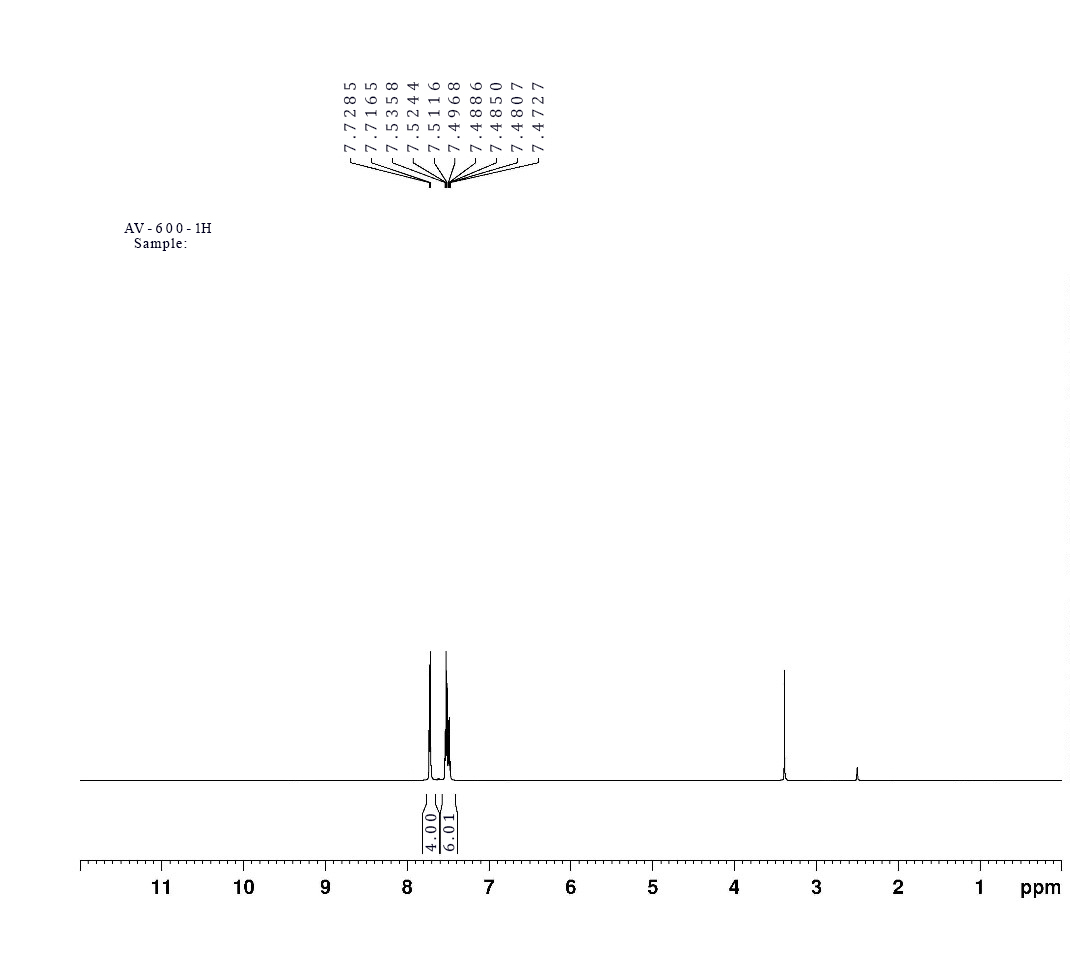


^1^H NMR Spectrum of sulfinyldibenzene


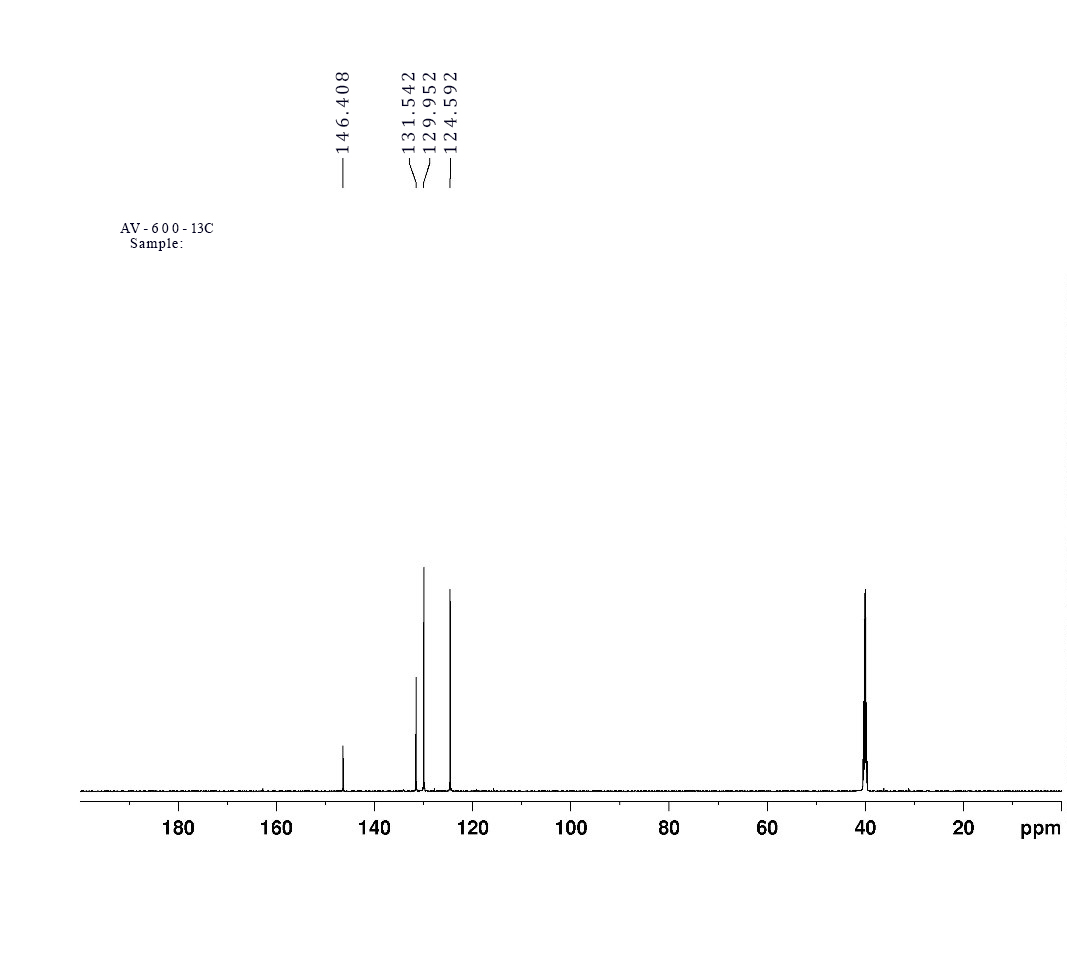


^13^C NMR Spectrum of sulfinyldibenzene
